# Expression of miR-1, miR-133a, miR-133b and miR-206 increases during development of human skeletal muscle

**DOI:** 10.1186/1471-213X-11-34

**Published:** 2011-06-07

**Authors:** Andrie Koutsoulidou, Nikolaos P Mastroyiannopoulos, Denis Furling, James B Uney, Leonidas A Phylactou

**Affiliations:** 1 Department of Molecular Genetics, Function & Therapy, The Cyprus Institute of Neurology & Genetics, P.O. Box 2346, 1683 Nicosia, Cyprus; 2 UPMC Univ Paris 06, UM76, Institut de Myologie, and INSERM, U974 and CNRS, UMR7215, Paris, France; 3 The Henry Wellcome Laboratories for Integrative Neuroscience and Endocrinology, Dorothy Hodgkin Building, University of Bristol, Whitson Street, Bristol BS1 3NY, UK

## Abstract

**Background:**

MicroRNAs (miRNAs) are small RNA molecules that post-transcriptionally regulate gene expression and have been shown to play an important role during development. miR-1, miR-133a, miR-133b and miR-206 are expressed in muscle tissue and induced during muscle cell differentiation, a process that directs myoblasts to differentiate into mature myotubes, which are organized into myofibers. Although miR-1, miR-133a, miR-133b and miR-206 are well-studied in muscle, there is no information about their expression and function during human development. The purpose of this study was to determine the profile of these miRNAs in muscle cells isolated from different stages of human development.

**Results:**

We examined the levels of miR-1, miR-133a, miR-133b and miR-206 during the development of human foetus. All four miRNA levels were found increased during late stages of human foetal muscle development. Increases in the expression levels of these miRNAs were proportional to the capacity of myoblasts to form myotubes. Changes in miRNA levels during human foetal development were accompanied by endogenous alterations in their known targets and also in their inducer, MyoD. Ectopic MyoD expression caused an induction of muscle cell differentiation *in vitro*, accompanied by an increase in the levels of miR-1, miR-133a, miR-133b and miR-206.

**Conclusions:**

This study provides data about the profile of four miRNAs in human muscle cells isolated during different stages of foetal development. These results may shed light on the differentiation of muscle cells and regulation of muscle formation through miRNAs, during the development of human foetus.

## Background

MicroRNAs (miRNAs) are small (~22 nucleotides) non-coding RNAs that negatively regulate gene expression at the post-transcriptional level [1,2]. miRNAs bind to the 3' untranslated region (3'UTR) of their target mRNAs, causing either inhibition of protein translation or mRNA degradation [2]. During the last few years, intense research has revealed the regulatory role of miRNAs in almost all cellular processes, during health and disease [3-6].

Skeletal myogenesis is a complex and tightly regulated developmental process that directs myoblasts to differentiate into mature myotubes. During myogenesis, myoblasts are stimulated to initiate the expression of myogenic differentiation-specific genes, withdraw from cell cycle and fuse together to form multinucleated myotubes, which are ultimately organized into myofibers [7]. Myogenesis occurs during embryogenesis in order to form muscle and in adults to replace lost or damaged muscle. Most embryonic skeletal myogenic progenitors arise from somites, which are masses of paraxial mesoderm distributed along the two sides of the neural tube [8]. Somites differentiate along the dorsal-ventral axis to give rise to the dorsally located epithelial dermomyotome and the ventrally located mesenchymal sclerotome [8]. The dermomyotome gives rise to musculature and dermis, whereas the sclerotome develops into the cartilage and bone. Myogenic precursors confined in the epithelium of the dermomyotome express Pax3, Pax7 and low levels of the myogenic determination factor Myf-5. Delamination of muscle progenitor cells from the dermomyotome causes the formation of the myotome, the third somitic compartment, which contains the first differentiated myofibers [9]. During the late stages of embryogenesis, a specialized population of myogenic stem cells, termed satellite cells, arise in order to provide most of the myonuclei to adult muscles during the postnatal growth of muscle tissue [8].

There has been an ever growing number of miRNAs found to be expressed in muscle tissue. Of these, the most extensively studied are miR-1, miR-133 and miR-206. miR-1 and miR-133 are highly expressed both in skeletal and cardiac muscles, whereas miR-206 is specifically expressed only in skeletal muscle [10,11]. In human and mouse, these miRNAs are encoded by three loci, each of which produces a bicistronic transcript, containing one miRNA from the miR-1/206 family and one from the miR-133 family (miR-133a, miR-133b) [10].

All four muscle miRNAs are induced during muscle differentiation, implying a critical role in the regulation of the process [10,11]. The expression of these miRNAs was found to be regulated by key myogenic regulatory factors (MRFs), including myogenic differentiation 1 (MyoD), myogenin [12], myocyte enhancer factor 2 (MEF2) [13] and serum response factor (SRF) [10]. Although miR-1, miR-133 and miR-206 are related in terms of expression, they have different targets, biological functions and transcriptional activation [4,10,14-17]. Cell culture experiments have shown that miR-1 and miR-206 promote muscle cell differentiation, whereas miR-133 promotes cell proliferation by down-regulation of different target genes [10,11].

Currently there is very little information about the expression and function of muscle-specific miRNAs in development and specifically in human development. The purpose of this study was to investigate the expression of miR-1, miR-133a, miR-133b and miR-206 at different stages of the human developing muscle and during differentiation in myoblast cell lines. The levels of all four miRNAs were found proportional to the stage of muscle development. Moreover, all four miRNAs were found elevated during *in vitro* differentiation of myoblasts to myotubes. Increases in the levels of these miRNAs were accompanied by alterations in their known targets and also in their inducer, MyoD. Finally, overexpression of MyoD in these cells caused an increase in muscle cell differentiation *in vitro *and an induction of muscle miRNA gene expression.

## Results and discussion

### Human myoblasts from later stages of development have an increased capacity to differentiate

**Figure 1:**
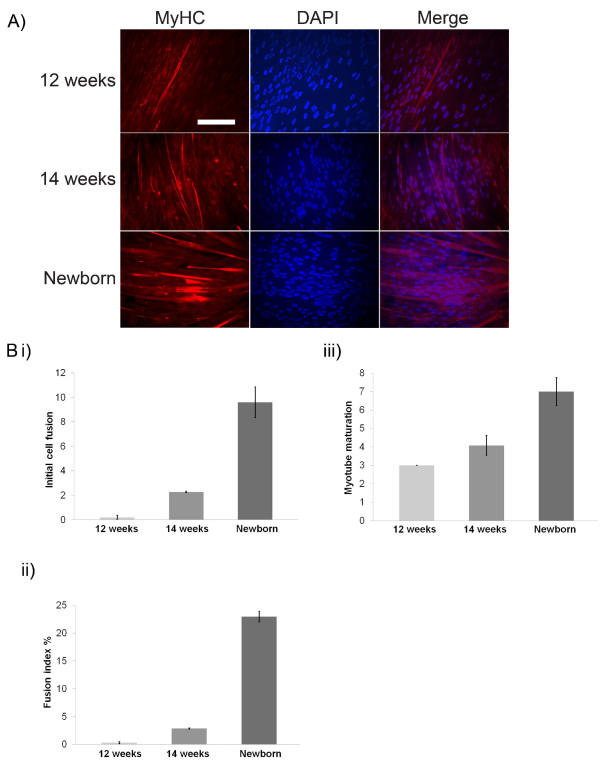
**Muscle cell differentiation increases during the late stages of foetal development**. (A) Muscle cell differentiation was detected in all three human myoblast cell lines (12-week old, 14-week old and newborn) by immunocytochemistry against myosin heavy chain (MyHC). DAPI was used to stain cell nuclei. Scale bar, 0.8 mm. (B) Muscle formation was characterized by measuring or calculating (i) the average number of myotubes per area (initial cell fusion), (ii) the percentage of nuclei present in myotubes compared to the total number of nuclei present in each area (fusion index %) and (iii) the average number of nuclei per myotube (myotube maturation) in total 10 areas of cells.

Although there are many reports regarding human muscle development, there is limited information about the capacity of isolated myoblasts to differentiate *in vitro *into mature myotubes during different stages of muscle foetal development [18]. The capacity of myoblasts, isolated from different stages of the development of human foetus to differentiate into myotubes was first examined. For this study, human myoblasts were used from cell lines of three different developmental stages of the foetus (12-week old human foetus, 14-week old human foetus and newborn). Following *in vitro *differentiation induction, myoblasts isolated from 12-week old human foetus showed very little myotube formation (Figure 1A). Similar results were obtained with 14-week old foetal myoblasts even if a slightly increased capacity to form myotubes was observed when compared to 12-week old myoblasts. In contrast, myoblasts isolated from newborn displayed the highest capacity to form mature multinucleated myotubes. Muscle cell differentiation was further characterised and proved that formation and maturity of myotubes was significantly higher in the newborn cell line compared to the foetal cell lines (Figure 1B).

### miR-1, miR-133a, miR-133b and miR-206 levels are proportional to the stage of muscle development

**Figure 2:**
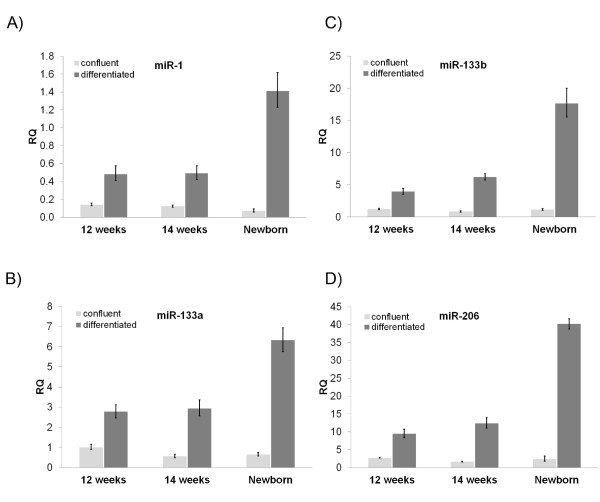
**Muscle miRNA expression levels increase during muscle foetal development**. 12-week and 14-week old foetal and newborn human myoblasts were grown to confluency or induced to differentiate for 5 days. miR-1 (A), miR-133a (B), miR-133b (C) and miR-206 (D) levels were significantly higher in newborn myotubes compared to foetal myotubes. miRNA levels were found to be low in confluent cells.

There is currently no existing evidence about the expression of miR-1, miR-133a, miR-133b and miR-206 genes during the stages of human muscle development. miRNA levels were compared between the three myoblast cell lines from different stages of human foetal development (12-weeks, 14-weeks and newborn). Each of the four miRNAs showed a similar expression pattern in the three stages of muscle development, which is probably due to a similar mechanism of regulation. There was a steep increase in all four miRNA levels in cells isolated from the newborn, compared to cells isolated from 12-week and 14-week old foetuses (Figure 2). Moreover, there was a slight increase in the miRNA levels in cells isolated from the 14-week old foetus, compared to those of the 12-week old foetus (Figure 1 and Table 1). These data are consistent with previous findings that miR-1 and miR-133 are expressed in very small amounts in the developing heart and skeletal muscle of embryonic day 13.5 (E13.5) and E16.5 in mice and their expression increases in neonatal heart and skeletal muscle [10]. A similar observation regarding the increase of muscle-specific miR-206 during mouse embryonic development was recently reported [19]. Furthermore, these data agree with findings in zebrafish, showing that most miRNAs are expressed relatively late during embryogenesis [20].

**Table 1:** Statistical analysis of muscle miRNA expression levels during muscle foetal development

		miR-1 ΔCt	miR-133a ΔCt	miR-133b ΔCt	miR-206 ΔCt
Comparison between confluent and differentiated cells	12 weeks	p < 0.05	p < 0.05	p < 0.05	p < 0.05
	
	14 weeks	p < 0.05	p < 0.05	p < 0.05	p < 0.05
	
	Newborn	p < 0.05	p < 0.05	p < 0.05	p < 0.05

Comparison between confluent and differentiated cells (all developmental stages combined)		p < 0.0001	p < 0.0001	p < 0.0001	p < 0.0001

Comparison between developmental stages (differentiated cells)		p < 0.05	p < 0.05	p < 0.005	p < 0.005

Comparison of miRNA levels between confluent and differentiated muscle cells at each developmental stage or combined was performed. Comparison of miRNA levels between developmental stages of differentiated muscle cells was carried out. All results were statistically significant (p-value < 0.05).

miR-1, miR-133a, miR-133b and miR-206 were found to be expressed during muscle cell differentiation both in adult primary human myoblasts and adult mouse cell lines [10-12]. Isolated myoblast cell lines were examined for the induction of these miRNAs during differentiation. miR-1, miR-133a, miR-133b and miR-206 levels were low in undifferentiated myoblasts, signifying that they are not highly expressed during the stages before differentiation (Figure 2). On the contrary, when cells were induced to differentiate *in vitro*, a marked increase was observed in the levels of all four miRNAs, suggesting their active role in the process (Figure 2). These results agree with previous work performed on adult mouse cell lines and adult primary human myoblasts [10-12].

The expression patterns of miRNAs in the various stages of muscle development are proportional to the differentiation levels seen in these cells. It can be therefore assumed that miR-1, miR-133a, miR-133b and miR-206 levels correlate with the induced *in vitro *differentiation of myoblasts to myotubes. The relationship between the four miRNAs and differentiation, during human development is analogous to that shown in adult human and mouse muscle cells [10-12].

### Alterations in muscle-specific miRNA known targets and inducer levels during human muscle development

Although miR-1, miR-133a, miR-133b and miR-206 genes have similar expression patterns, they have different targets and biological functions [4,10]. Among the four miRNAs, miR-1 and miR-206 were found to promote muscle cell differentiation [10,11]. miR-1 was shown to bind to the 3'UTR of the histone deacetylase 4 (HDAC4), an inhibitor of muscle differentiation, and suppresses its expression during growth and differentiation conditions [10,21,22]. Connexin 43 (Cx43), a gap junction channel required in embryonic skeletal muscle, which is also down-regulated during late embryogenesis and early post-natal life was found to be an experimentally verified target of miR-206 and miR-1 during myogenesis [15].

**Figure 3:**
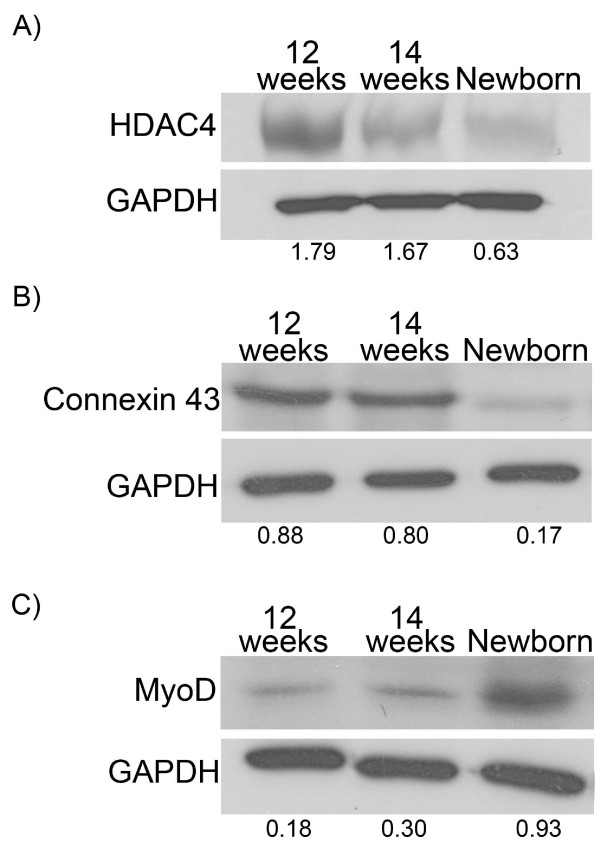
**Alterations in miRNA targets and inducer in muscle cells during development**. Western blot analysis showed increased levels of HDAC4 (A), a verified target of miR-1, and Connexin 43 (B), a verified target of miR-206 and miR-1 in foetal muscle cells, compared to newborn cells. Western blot analysis of MyoD (C), an inducer of all four miRNAs, showed decreased levels in foetal muscle cells compared to newborn cells.

Protein analysis showed a reduction in the protein levels of HDAC4 and Cx43 in newborn muscle cells compared to the two foetal cell lines (Figure 3A and Figure 3B). These results are analogous to the elevated miR-1 and miR-206 levels observed in the newborn muscle cell line in part 3.2 (Figure 2).

MyoD is a master myogenic transcriptional regulatory factor that activates a number of muscle-specific genes to drive muscle cell differentiation [23]. It is required at initial stages of myogenesis for the commitment of multipotential somite cells to the myogenic lineage [24]. MyoD is also expressed in proliferating myoblasts thus regulating the transition from proliferation to differentiation [25]. Experiments on adult mouse C2C12 and mouse embryonic fibroblasts showed that MyoD binds to regions upstream of miR-1, miR-133a and miR-206 and regulates their expression [12,14]. As expected, MyoD levels were elevated in the newborn cell line, compared to the foetal cell lines (Figure 3C). This finding comes into agreement with the observations that newborn myoblasts can form more myotubes and have elevated miRNA levels (Figure 1 and Figure 2).

### Restoration of decreased MyoD levels promotes muscle cell differentiation *in vitro *and increases miR-1, miR-133a, miR-133b and miR-206 gene expression in human foetal myoblasts

Forced expression of MyoD in non-muscle cells in culture can induce myogenic differentiation, whereas *MyoD*-null primary myoblasts exhibited reduced differentiation [26,27].

**Figure 4:**
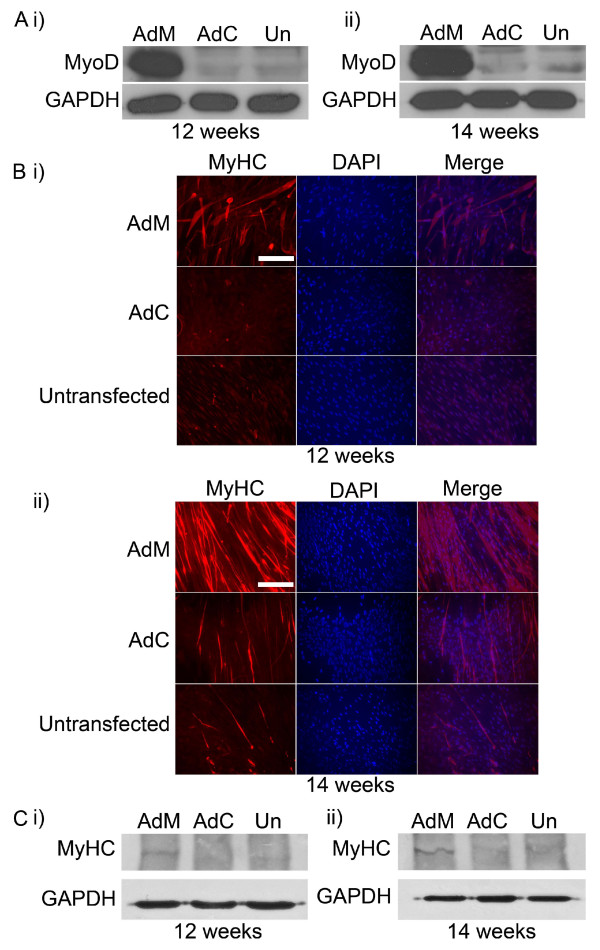
**MyoD induces myotube formation in human foetal muscle cells**. (A) Overexpression of endogenous MyoD levels were detected by Western blotting in 12-week old (i) and 14-week old (ii) muscle cells, transfected with the MyoD adenovirus (AdM), compared to adenovirus-control transfected cells (AdC) and untransfected cells (Un). (B) Immunocytochemistry of the myogenic marker myosin heavy chain (MyHC) showed a marked increase in myotube formation in cells overexpressing MyoD, compared to control cells. Nuclei were stained with DAPI. Scale bar, 0.8 mm. (C) Overexpression of MyoD resulted in increased levels of MyHC levels, as detected by Western blotting, in 12-week old (i) and 14-week old (ii) muscle cells, compared to control cells.

Foetal cell lines displayed low levels of muscle cell differentiation, accompanied by low expression levels of MyoD and miRNAs (Figures 1, 2 and Figure 3C). In order to induce muscle cell differentiation in 12-week and 14-week old foetal myoblast cell lines and determine whether miRNA levels will also be stimulated, cells were transfected with an adenovirus expressing the MyoD transcription factor (AdM) (Figure 4A). *In situ *analysis of myotube formation showed an increase in their ability to form myotubes in both 12-week and 14-week old foetal muscle cell lines in MyoD-transfected cells, compared to control cells (Figure 4B and Figure 4C).

**Figure 5:**
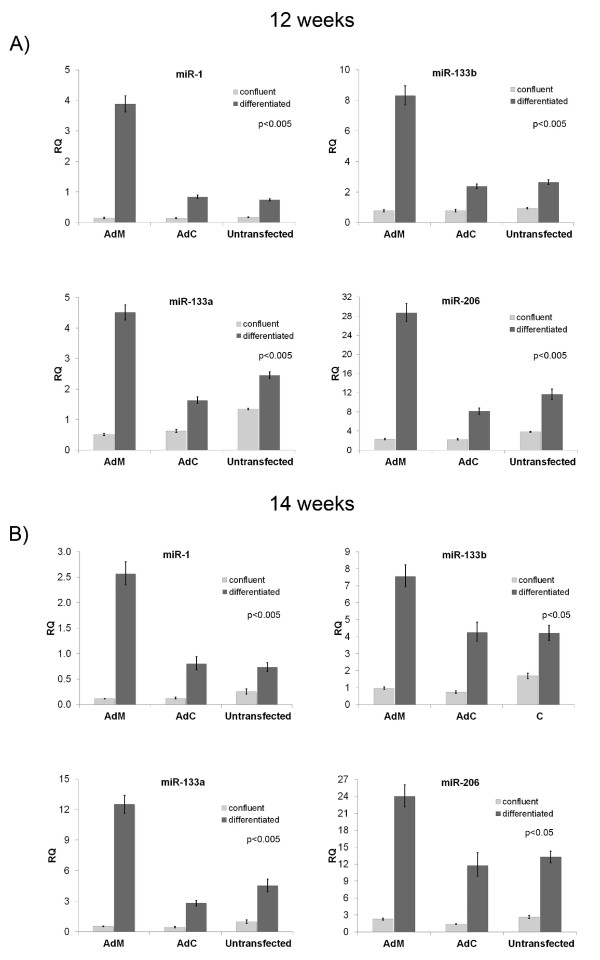
**MyoD induces miRNA levels in human foetal muscle cells**. A significant increase in the levels of all four miRNAs was detected by Real-Time PCR in 12-week old (A) and 14-week old (B) MyoD-transfected muscle cells, following differentiation, compared to control-transfected and untransfected cells. Statistical analysis comparing the differentiated AdM, AdC and untransfected miRNA levels revealed significant differences with the corresponding p-values indicated in each graph.

Levels of miR-1, miR-133a, miR-133b and miR-206 were next investigated in MyoD-transfected cells. Following differentiation, all four miRNAs were found significantly increased in MyoD-transfected cells from both cell lines, compared to control cells (Figure 5).

These results suggest a mechanism by which MyoD induces *in vitro *muscle cell differentiation in human foetal cells, accompanied by the induction of miR-1, miR-133a, miR-133b and miR-206 levels *in vitro*.

## Conclusions

Although miR-1, miR-133a, miR-133b and miR-206 have been extensively studied, there is no information about their expression during the development of human skeletal muscle. Results presented in this study show that miR-1, miR-133a, miR-133b and miR-206 are induced during human muscle cell differentiation and their levels are increased proportionally to the stage of muscle foetal development. Since miRNAs regulate important processes during development, such information would be very important for understanding the mechanism of muscle formation during foetal development. Results presented in this work can provide insights of muscle cell differentiation during the development of human foetus and offer valuable information about the implication of miRNAs in congenital myopathies.

## Methods

### Human skeletal muscle cell culture and adenovirus transfections

Human myoblasts were isolated under sterile conditions from muscle biopsies which showed no signs of neuromuscular disease. The biopsies were obtained from the Tissue Bank for Research (BTR) of the Association Francaise contre les Myopathies in accordance with the French legislation on ethical rules and the local Bioethics Committee. For foetuses (12 and 14 weeks of development), biopsies were taken from the proximo-ventral region of the limb, which gives rise to the quadriceps [18]. For the newborn infant (5 days after birth), the biopsy was taken from the quadriceps [28]. The stage of development was calculated for each foetus at the autopsy and based on morphometric analysis of the head, femur and foot. For the infant, the age was calculated from birth.

Myoblasts were grown in Dulbecco's Modified Eagle Medium (DMEM) (Invitrogen) supplemented with 20% Foetal Bovine Serum (FBS) (Invitrogen) (growth medium). Medium was changed every day. When cells reached confluency, they were differentiated with DMEM supplemented with 2% Horse Serum (Invitrogen) (differentiation medium) for 5 days or processed as mentioned elsewhere. In the case of MyoD adenovirus transfections, 2 days before confluency, myoblasts were transfected with 100 M.O.I. of human MyoD adenovirus (Vector Biolabs) (AdM) or control virus (Vector Biolabs) (AdC), expressing a non-specific target, left to grow to confluency and then induced to differentiate or processed as explained elsewhere.

### miRNA analysis

Total RNA enriched with small RNAs, including miRNAs, was extracted from muscle cells at confluency or after 5 days of differentiation, using mirVana™ miRNA Isolation Kit (Ambion). The extracted RNA was subjected to Reverse Transcriptase PCR using the TaqMan^® ^MicroRNA Reverse Transcription Kit (Applied Biosystems). miRNA levels were measured by Real-Time PCR amplification using TaqMan^® ^MicroRNA Assays specific for miR-1, miR-133a, miR-133b and miR-206 (Applied Biosystems), according to the manufacturer's instructions. miRNA expression was normalized to the RNA U6B small nuclear (RNU6B) (Applied Biosystems).

### Immunocytochemistry

Differentiated cells were fixed in 4% parafolmaldehyde and incubated with a monoclonal MY32 antibody against myosin heavy chain (MyHC) (Sigma) and a Texas-red-conjugated anti-mouse secondary antibody (Jackson Laboratories). Nuclei were stained with DAPI (Vysis). Images were captured with a NIKON digital camera and then assembled using Adobe Photoshop software. Myotubes were counted three times for each cell line from ten different areas of cells.

### Western Blotting

Confluent myoblasts or differentiated muscle cells were subjected to protein extractions. 40 - 60 μg of protein extracts were incubated with HDAC4 (1:200, Santa Cruz), Connexin 43 (1:200, Santa Cruz), MyoD (1:300, Santa Cruz), MyHC (1:300, Sigma) or GAPDH (1:1500, Santa Cruz) primary antibodies, followed by incubation with goat anti-mouse IgG or donkey anti-rabbit IgG secondary antibodies conjugated to horseradish peroxidase (Santa Cruz).

### Statistical analysis

Statistical analysis was performed as described before [29], using SAS, v 9.1 (SAS Institute Inc., Cary, NC, USA) software. ΔCt values were calculated as the Ct(miRNA) - Ct(RNU6B). Exact Wilcoxon tests were used to compare the ΔCt parameters. Analyses were performed both at each developmental stage (12 weeks, 14 weeks and newborn) and for all stages combined. A p-value of below 0.05 was considered statistically significant. All probabilities were two-tailed.

## Authors' contributions

AK carried out human skeletal muscle cell culture, adenovirus transfections, miRNA analysis, immunocytochemistry, western blotting and drafted the manuscript. NPM participated in immunocytochemistry. DF isolated myoblasts, established cell lines and participated in project design. JBU participated in project design. LAP conceived the study and participated in its design and coordination. All authors read and approved the final manuscript.
